# Predicting Dynamic Clinical Outcomes of the Chemotherapy for Canine Lymphoma Patients Using a Machine Learning Model

**DOI:** 10.3390/vetsci8120301

**Published:** 2021-12-02

**Authors:** Jamin Koo, Kyucheol Choi, Peter Lee, Amanda Polley, Raghavendra Sumanth Pudupakam, Josephine Tsang, Elmer Fernandez, Enyang James Han, Stanley Park, Deanna Swartzfager, Nicholas Seah Xi Qi, Melody Jung, Mary Ocnean, Hyun Uk Kim, Sungwon Lim

**Affiliations:** 1 ImpriMed, Inc., 4030 Fabian Way, Palo Alto, CA 94303, USA or jamin@imprimedicine.com (J.K.); peterklee@imprimedicine.com (P.L.); apolley@imprimedicine.com (A.P.); prsumanth@imprimedicine.com (R.S.P.); jgtsang@imprimedicine.com (J.T.); fernandez@imprimedicine.com (E.F.); hanenyang@gmail.com (E.J.H.); stanleyp@imprimedicine.com (S.P.); dmswartzfager@gmail.com (D.S.); sxq.nicholas96@gmail.com (N.S.X.Q.); melody@imprimedicine.com (M.J.); mary@imprimedicine.com (M.O.); 2 ImpriMedKorea, Inc., Seoul Startup Hub, Seoul 04147, Korea; chbr972@imprimedicine.com; 3 Department of Chemical Engineering, Hongik University, Seoul 04066, Korea; 4 Department of Chemical and Biomolecular Engineering, Korea Advanced Institute of Science and Technology (KAIST), Daejeon 34141, Korea; ehukim@kaist.ac.kr

**Keywords:** lymphoma, machine learning, chemotherapy, precision medicine

## Abstract

First-line treatments of cancer do not always work, and even when they do, they cure the disease at unequal rates mostly owing to biological and clinical heterogeneity across patients. Accurate prediction of clinical outcome and survival following the treatment can support and expedite the process of comparing alternative treatments. We describe the methodology to dynamically determine remission probabilities for individual patients, as well as their prospects of progression-free survival (PFS). The proposed methodology utilizes the ex vivo drug sensitivity of cancer cells, their immunophenotyping results, and patient information, such as age and breed, in training machine learning (ML) models, as well as the Cox hazards model to predict the probability of clinical remission (CR) or relapse across time for a given patient. We applied the methodology using the three types of data obtained from 242 canine lymphoma patients treated by (L)-CHOP chemotherapy. The results demonstrate substantial enhancement in the predictive accuracy of the ML models by utilizing features from all the three types of data. They also highlight superior performance and utility in predicting survival compared to the conventional stratification method. We believe that the proposed methodology can contribute to improving and personalizing the care of cancer patients.

## 1. Introduction

Cancer heterogeneity has been extensively reported and studied over the past five decades. The findings have enabled classification and categorization of tumors into subtypes sharing, for example, the same molecular features such as the overexpression of antigens. Advances in subtyping have resulted in the improved care and prognosis of cancer patients as therapies tailored to the subtypes have proven to be more effective than unanimously administered ones [1,2,3]. Perhaps one of the most well-known achievements of tailored (namely, precision) medicine is the drastic improvement in survival of the human epidermal growth factor receptor (HER) positive patients treated with trastuzumab, an antibody targeting this receptor [4].

A wide variety of factors, including physical, anatomical, radiographic, genetic, and histological features, are considered when analyzing cancer heterogeneity. Inspired by the latest advances in artificial intelligence (AI) that are effective in dealing with complex systems, researchers began to apply AI, especially machine learning (ML) techniques, to subtyping cancer. Pioneering attempts began by using a single category of data. McCarthy et al., for example, used the visualization and classification technique to differentiate lung cancers with respect to the selected set of genes [5]. Recent advances in computing power have enabled the processing of more complex data such as radiographic images. Zhou et al. developed computational image descriptors to assist in extracting features from magnetic resonance imaging (MRI) results that are used to characterize brain tumors [6]. The latest work in the field began to employ combinations of different data such as MRI and genomics for subtyping tumors as well as predicting survival, with the end goal of assisting and improving treatment choices [7,8,9]. 

Towards this aim, we previously reported the development of the ML approach for predicting in vivo response to a single chemotherapeutic drug [10]. Random forest (RF) models were trained using ex vivo drug sensitivity analysis and flow cytometry results to estimate the probability of positive response to a given drug. Our work was in part motivated by one of the pioneering works by Shipp and colleagues where they employed a supervised ML to classify diffuse large B-cell lymphoma patients into subgroups featuring distinct overall survivor rates based on the gene expression profiles [11]. A more personalized technology predicting drug responses of individual cancer patients was recently developed by Mucaki et al. [12]; the technology also relied on gene signatures as features for the ML models, which predicted remission by a platin agent with as high as 72% accuracy. Non-genomic data ranging from magnetic resonance imaging to histology are also used successfully in developing the ML-based predictive models [7,13,14,15,16]. While most models outperformed human experts to a varying degree, they were focused only on long-term survival and/or clinical outcome at a long run, i.e., 3 to 5 years after chemotherapy. To our best knowledge, no models have been reported that predict the patient’s responses across multiple time points. Such prospects can be especially helpful when deciding whether or not to continue with the current treatment for those showing no (immediate) responses.

In this study, we propose a novel methodology for predicting dynamic clinical outcomes and survival of cancer patients treated with a first-line chemotherapy. The proposed methodology is applied using the data obtained from canine lymphoma patients who received (L-)CHOP chemotherapy. Three different types of data were utilized to train ML models that generate a probability of clinical remission (CR) by the various time points; several types of ML methods are employed to demonstrate how the predictive accuracy varies among these methods. The same data are also used to develop a Cox hazards model for predicting the progression-free survival (PFS) of each patient. We then illustrate how the trained model enhances stratification of the patients when analyzing PFS.

## 2. Materials and Methods

### 2.1. Case Selection

A total of 242 were chosen from the pool of canine lymphoma patients who had received the service by ImpriMed, Inc. (Palo Alto, CA, USA). All the patient samples were collected under the informed consent forms approved by the internal review boards (IRB) and ethical committee of the participating veterinary hospitals. Chemotherapy was administered based on the standard operating protocols managed by board-certified veterinary oncologists. The case selection began by screening the patients who received at least 3 of the 4 or 5 drugs that constitute (L-)CHOP chemotherapy within the first four weeks of diagnosis. We then selected the subset that met the following three conditions: (1) the availability of 70% or more of the drug sensitivity (DS) and flow cytometry (FC) data; (2) a prognosis of at least the first 12 weeks since the administration of chemotherapy; (3) the availability of age, sex, breed, and at least 25% of the rest of the patient information (PI) data. The detailed information on the demographic of the selected subgroup is provided in Table 1.

Further information such as the distribution with respect to albumin is provided in Figure S3.

### 2.2. The Development of the Predictive ML Models

Categorical data such as breed, sex, and subtype were converted into numerical values by the label encoding technique [17]. All numerical data were rescaled using the robust scaler method [18]. The missing data were replaced with the median unless stated otherwise; this was achieved after converting categorical data into numerical data and rescaling them. As explained in the main text, we tried replacing the missing data with imputed values. Imputation was performed in two ways—*k* nearest neighbors with *k* = 5 and the MICE (multivariate imputation by chained equation) technique [19,20].

The compiled data were used to develop three ML models—random forest, support vector machine, and linear regression. For each, the data were randomly split into train set and test set (3:1) in a stratified manner to preserve the ratio of the outcomes in both sets [18]. When the FC and/or PI data were used in addition to the DS data as features, a *k* subset was chosen with respect to mutual information [21]. Hyperparameters for each model were tuned by creating a grid (Table S4) and searching for the combination that resulted in the optimal performance. The outputs of ML models (classifiers) were the probabilities of achieving CR by the 4th, 8th, or 12th week since the administration of chemotherapy. The importance of features was assessed only for the best predictive models (RF) using the mean decrease in node impurity [22].

### 2.3. Survival Analysis

The progression-free survival over time for each patient was predicted by utilizing the Cox proportional hazards model. Starting with the same set of features used in the ML models, we removed several features that restricted the convergence. The prognosis and duration of the PFS for each patient included in this retrospective study were retrieved from the medical records provided by the veterinary hospitals. The categorical data were transformed into numerical values using one hot encoder and then scaled via removing the mean and scaling to unit variance. The patient stratification for the survival analysis was performed with respect to the predicted duration of the period having the probability of relapse lower than 50%. The patient was classified as high or low when the predicted duration was longer or shorter than the subtype’s average, respectively.

### 2.4. Model Performance Assessment

The performance of the three ML models predicting clinical outcome across the time points was first compared with respect to the average of the area under the receiver operating characteristic curve (ROC-AUC) and accuracy from the 4-fold cross-validation. We next evaluated the positive predictive value (PPV), negative predictive value (NPV), sensitivity, and specificity of the chosen models to better understand the predictive ability. Prism 8 (GraphPad, San Diego, CA, USA) and R Studio (v1.74) were used to perform statistical analyses and create graphs. The *p* values shown in the graphs are calculated using the unpaired *t* or log-rank test.

## 3. Results

### 3.1. Data Structure, Acquisition, and Model Training

**Figure 1:**
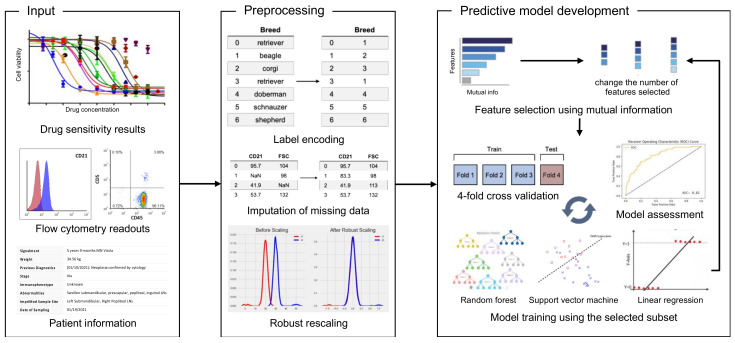
The three types of data used in the ML models and schematic overview of the proposed methodology for predicting dynamic clinical outcomes of canine lymphoma patients treated with (L-)CHOP chemotherapy.

Three types of data—drug sensitivity (DS), flow cytometry (FC), and patient information (PI)—were obtained for each lymphoma patient included in this study (Figure 1). The baseline characteristics of all 242 patients are summarized in Table S1. Experimentally measured IC_50_ and maximum cytotoxicity of five drugs that constitute the (L-)CHOP chemotherapy—L-asparaginase, vincristine, cyclophosphamide, doxorubicin, and prednisone—were included in the DS data, as well as those of eight other chemotherapeutic drugs that can be used to treat canine lymphoma (Table S1). Nine flow cytometry parameters involving cancer cell size, shape, and antigen expression distributions were selected for use in training the machine learning models. Patient information consisting of 33 features (Table S2 shows the full list) such as age, sex, breed, and bloodwork were extracted manually from the reports submitted by veterinary oncologists. In this manner, we obtained a total of 70 features for each of the 242 patients included in this retrospective study.

**Figure 2:**
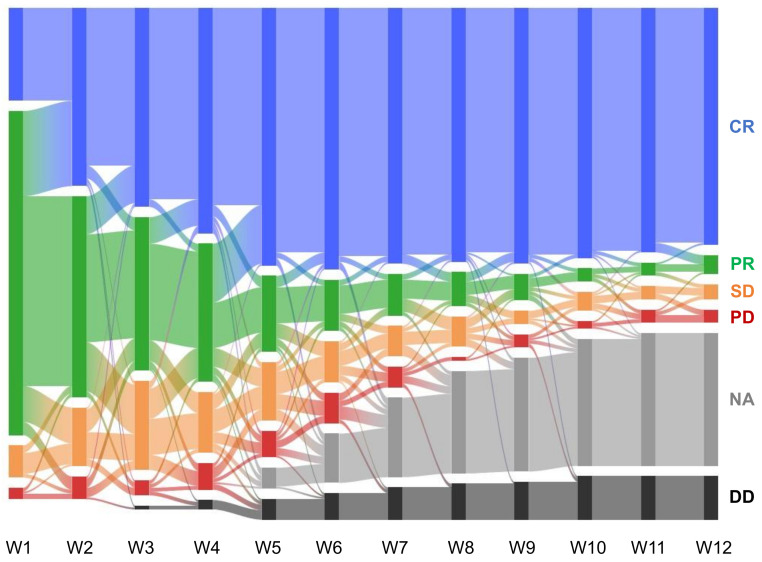
Dynamic changes in the clinical outcomes of the patient cohort during the first 12 weeks of the (L-)CHOP chemotherapy. CR, PR, SD, and PD denote clinical remission, partial response, stable disease, and progressive disease reported by the vets. “NA” represents the cases where the patients were no longer treated with the (L-)CHOP chemotherapy, while “DD” (dead) includes the cases where the patients were euthanized.

Canine lymphoma patients treated with the (L-)CHOP chemotherapy typically undergo a 15 weeks-long treatment [23]. The clinical outcomes (classified as complete remission, partial response, stable disease, or progressive disease) of the patients during the first 12 weeks were first collected and analyzed. The aggregate data (Figure 2) showed that more than 98% of the patients who eventually achieved clinical remission achieved remission by the 8th week. Additionally, a significant portion (10%) of the patients stopped receiving (L-)CHOP chemotherapy because they had failed to achieve remission by the 4th week. Given these observations, we trained the machine learning models to predict the likelihood of achieving and remaining in clinical remission due to (L-)CHOP chemotherapy by the 4th, 8th, and 12th week; classifiers were used to predict whether or not a given patient will be in remission or not. For each time point, three different types of ML models—random forest (RF), support vector machine (SVM), and linear regression (LR)—were trained and tested to compare and identify the model exhibiting the optimal performance. These three models were chosen as they are routinely used in classification problems across fields.

### 3.2. Model Performance Based on DS and FC Data

**Figure 3:**
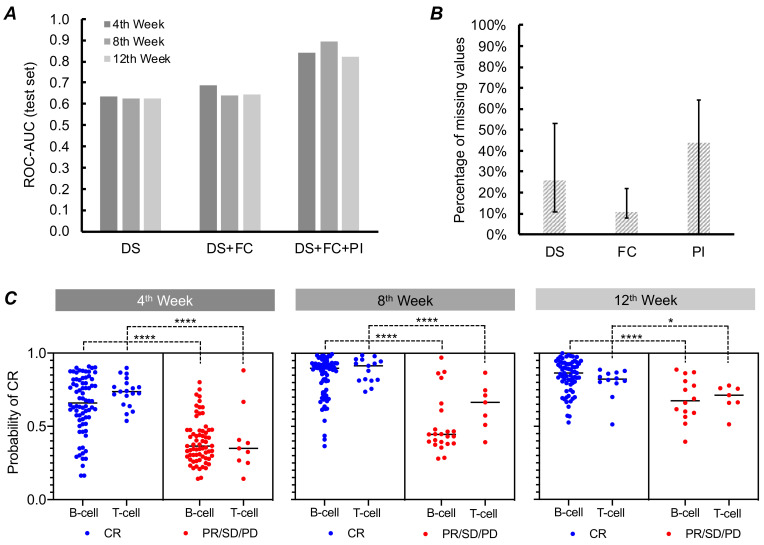
Predicting clinical outcome of the (L-)CHOP chemotherapy using the three types of data. (**A**) Comparison in the performance of the RF models in terms of ROC-AUC with different data sets across all time points. (**B**) Proportions of the missing values in each type of data. The error bars represent the minimum and maximum values observed within the features comprising the given data type. (**C**) Distribution of the probabilities of the positive clinical outcome generated by the RF models. The blue and red colored dots represent the values predicted for the patients who achieved or failed to achieve CR by the given time point, respectively. Asterisks represent significance levels (**** *p* < 0.0001; * *p* < 0.05).

We first trained the models using only the DS values to understand how the other types of data (FC and PI) contribute to improving model performance. From a total of 28 features representing IC_50_ and maximum cytotoxicity of the 14 chemotherapeutic drugs, we first trained an RF model using the DS of the five chemotherapeutic drugs constituting the (L-)CHOP chemotherapy. The ROC-AUC of the test set were 0.635, 0.624, and 0.626 when predicting the likelihood of CR for the 4th, 8th, and 12th week, respectively (Figure 3A). When allowed to choose the top ten features with respect to mutual information based on the nearest neighbors method [24], the performance improved slightly only for the model predicting the likelihood of CR by the 4th week. Similar levels of performance were observed when SVM and LR were used instead of the RF.

### 3.3. Model Performance Based on DS, FC, and PI Data

We hypothesized that PI data such as age, sex, blood cell levels, and biochemical concentrations could improve the predictive performance of the ML models by providing additional information not reflected in DS or FC data. As mentioned before, we examined medical records and extracted 33 features (Table S2). Unlike the other types of data (DS and FC) that are measured on site experimentally using the live tumor cells derived from the patients (Methods), the PI data relied solely on the records provided by the veterinary hospitals. Extensive resources were thus needed for retrieving data, mainly owing to the heterogeneity in report formats and diction across the hospitals and oncologists. More importantly, we were not able to obtain records for a substantial portion (up to 64%) of the patients included in this study (Figure 3B).

The predictive accuracy of the ML models improved remarkably when the PI data were used in addition to DS and FC data (Figure 3A). The ROC-AUC of the test set became as high as 0.893, with an average increase of 0.195 across the three time points. The predictive performance was the highest when the top 14 features were chosen with respect to mutual information. For the missing values in the PI data, we tried mean and median of each feature, as well as the synthetic data imputed by the *k* nearest neighbors [19] or multivariate imputation [21]. The highest model performance was obtained when the missing values were replaced with the median during pre-processing. RF models exhibited the highest performance across all time points, followed by LR and SVM; the performance of the latter two were significantly lower than that of the RF, especially when predicting the clinical response for the 8th and 12th week (Table S3).

Analyzing B- vs. T-cell subtypes in (canine) lymphoma reveals the origin of cancer and provides insights on prognosis [16,25]. Given the clinical importance, we analyzed distribution of the probabilities of achieving and maintaining CR for each subtype generated by the best models. The results confirmed effective differentiation between the positive and negative clinical outcomes for both cell types across all time points (Figure 3C). In other words, the means of the probabilities were significantly lower (*p* < 0.0001 except for the 12th week) for the patients whose clinical outcomes were indeed negative (PR, SD, or PD) than the means of the probabilities for the patients who did achieve clinical remission by the selected time points. The predictive performance of the best ML model for each time point is summarized in Table 2.

### 3.4. Progression-Free Survival Prediction

**Figure 4:**
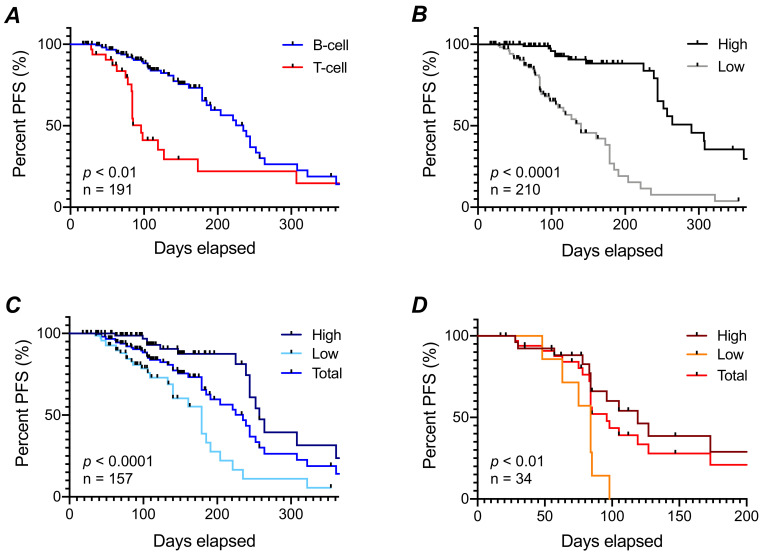
Application of the ML model in predicting prognosis. (**A**) Progression-free survival of B- vs. T-cell canine lymphoma patients. (**B**) The same PFS analysis based on the Cox model generated the number of days by which the probability of relapse reaches 50%; the discrepancies in n are due to some of the patients not being classified as either cell-type. The same PFS analysis using stratification based on the Cox model generated median days for the (**C**) B-cell and (**D**) T-cell subtypes among our cohort.

As is the case with human lymphoma patients, immunophenotypes and/or biomarkers have traditionally been used to project prospects of survival following the (L-)CHOP chemotherapy. The cell type—B- vs. T-cell—is a well-known conventional stratification method: The median duration of PFS is well documented: 244 days for the B-cell versus 108 days for the T-cell subtype [23,26,27]. We observed almost the same difference in prognosis between the two cell types among our cohort—235 vs. 96 days (Figure 4A). Given the predictive power of the ML models based on our database, we trained a Cox proportional hazard model to predict prognosis. The C-statistic became as high as 0.850 when all three types of data were used to train the model. The difference in prognosis was also more pronounced when comparing the PFS of the patients with respect to the median of PFS predicted by the trained Cox model (Figure 4B). The median was 290 versus 140 days for the high and low groups, respectively.

We were able to successfully combine the cell type and the prediction result generated by the Cox model to provide more precise predictions of survival. Among the B-cell lymphoma patients, the PFS was markedly higher for those predicted more favorably by the Cox model (Figure 4C). The median PFS was 267 versus 179 days, with 235 observed for all the B-cell patients included in this study. Similar results were obtained for the T-cell patients (Figure 4D) with a higher *p*-value, likely due to a low number of subjects (5-fold lower than that of the B-cell type). The median of the PFS was 119 versus 84 days, with 96 being the observed median for all the T-cell patients included. These results suggest that the proposed methodology can be used to provide a more precise survival model for individual patients.

## 4. Discussion

We developed the ML models for predicting clinical outcomes of canine lymphoma patients treated by (L-)CHOP chemotherapy, which is the standard treatment of choice based on previous clinical trials [23]. A total of 65–84% of the patients achieved remission in the trials [25,27]; 79% of the cohort included in this retrospective study achieved remission, excluding those whose treatment changed or stopped in the middle (Figure 2). Unlike in clinical trials with a fixed design, some of the patients received a modified version of the (L-)CHOP chemotherapy where one of the drugs was replaced with other drugs in the same therapeutic category. The most frequent case was an administration of mitoxantrone instead of doxorubicin, usually due to consideration of cardiotoxicity [28]. Owing to these moderate relaxations on the inclusion criteria, the cohort size (n = 242) in this study is the largest reported to date, especially for use in developing ML models for predicting clinical outcomes of combinatorial chemotherapy.

In the previous study, we successfully developed the ML model for predicting in vivo response of single chemotherapeutic drugs using DS and FC data [10]. We first tried to emulate the previous work by training ML models with the drug sensitivity of only five drugs that constitute the (L-)CHOP chemotherapy and flow cytometry readouts. The performance of these initial trials was not satisfactory (Figure 3A); the accuracies stayed below 0.70. We sought to improve the model performance and hypothesized that the efficacy of a combinatorial chemotherapy regimen like (L-)CHOP also depends on physiological conditions of patients’ whole bodies, not just of their cancer cells. We thus expanded the input data to the models by adding information about patients’ physical status described in the records provided by the veterinary oncologists. Since the patients in this retrospective study were from more than 50 veterinary hospitals across the U.S., we only extracted information commonly found across the heterogenous report types. Among the 33 features manually extracted, age, breed, and sex were provided in almost all of the reports. In contrast, we were able to obtain bloodwork and count results for only 35–64% of the patients. The performance of the model still improved drastically, with the highest accuracy nearing 0.90 when trained with these PI features in addition to the DS and FC data (Figure 3A). While this observation does not fully support our hypothesis, it proves that PI can help to make better predictions of cancer patients’ in vivo responses to chemotherapy. Henriques et al., for example, also reported the usefulness of peripheral blood ratios in predicting the prognosis of lymphoma patients [29].

The importance of features differed when predicting the probability of CR by the 4th, 8th, and 12th week of the (L-)CHOP chemotherapy. On average, the DS results of the drugs constituting the (L-)CHOP chemotherapy were more important when predicting the clinical outcome by the 4th week (Figure S2). The importance decreased for the 8th week, and they were not included in the top ten when predicting the outcome for the 12th week. This was somewhat expected since the DS data were measured using the live tumor cells obtained via FNA at the onset of the chemotherapy (0th week). The composition, state, and microenvironment surrounding the cancerous cells after the 7th or 11th week, for example, will likely be different from those of the sampling date [30,31,32]. In contrast, the importance of the PI data increased substantially when predicting the clinical outcome of the 12th week versus the earlier weeks. Age was the most important feature, followed by the hematocrit and red blood cell level, which are all part of the PI data. They were all reported to correlate with the prognosis of canine lymphoma patients [29,33].

Missing data are often detrimental to predictive accuracy of ML models [34,35,36]. In our ML models, they also resulted in lower performance. The accuracies of predicting clinical outcome for the 4th, 8th, and 12th week became at most several percent lower than the overall performance for each when at least three or more features were missing. Given their prevalence across features in our retrospective study, we tried several methods for handling missing data before use in ML models. Replacing them with the median resulted in the highest predictive performance; it was significantly better than replacing with the mean, likely owing to the skewed distribution of data for some features (Figure S3). Using the synthetic data imputed by the well-known algorithms was also not as successful as replacing with the median. We suspect that this is due to features such as blood cell counts and total protein levels having weak to insignificant correlations to the other types of data.

Overall, we believe that the proposed methodology and technology can contribute significantly to improving care for canine lymphoma patients. The likelihood of CR by the various time points provided by the proposed methodology can help make informed decisions. The individualized PFS prospects will also help the stakeholders to be better prepared for monitoring prognosis and planning follow-up visits after completion of the chemotherapy.

## 5. Patents

There is an ongoing patent application describing the use of functional data, genomic data, and patient information by a machine learning technique for predicting in vivo responses to chemotherapy.

## Figures and Tables

**Table 1:** Characteristics of the patients included in this retrospective study.

Parameter	CR Prediction Study Population (N = 242)	PFS Prediction Study Population (N = 210)
**Age**		
Median ± SD	9 ± 3.2	8 ± 3.2
Range	1 to 17 years	2 to 16 years
**Sex**		
Male	56%	57%
Female	41%	42%
Unknown	3%	1%
**Relapse vs. Naïve**		
Naïve	90%	93%
Relapse	6%	5%
Unknown	4%	2%
**Immunophenotype**		
B	71%	75%
T	15%	16%
Others	14%	9%
**Clinical Stage**		
2	1%	2%
3	40%	43%
4	21%	19%
5	6%	3%
Not Available	31%	33%

**Table 2:** Performance of the best ML models predicting clinical outcome of the canine lymphoma patients treated with the (L-)CHOP chemotherapy.

Metrics	4th Week	8th Week	12th Week
Accuracy	0.804	0.891	0.827
PPV	0.824	0.894	0.879
NPV	0.791	0.875	0.500
Sensitivity	0.816	0.971	0.879
Specificity	0.800	0.636	0.500

## Data Availability

The data that support the findings of this study are not publicly available due to privacy restrictions. Requests to access the datasets should be directed to the corresponding author.

## Supplementary Materials

The following are available online at https://www.mdpi.com/article/10.3390/vetsci8120301/s1, Figure S1: Changes in the predictive performance of the RF model when the number of selected features *k* are increased from 7 to all; Figure S2: Importance of the features to the ML models predicting clinical outcome by the 4th, 8th, and 12th week since the initiation of the chemotherapy; Figure S3: Distribution of the raw data for the features having high (>50%) percentages of missing values; Table S1: List of the eight additional chemotherapeutic drugs for canine lymphoma considered in this study; Table S2: List of the 33 features comprising the PI data; Table S3: Performance of the other ML models when predicting clinical outcome across the time points; and Table S4: Grid of hyperparameters used to optimize the performance of the RF models.
