# Supplementary material for: Predicting Dynamic Clinical Outcomes of the Chemotherapy for Canine Lymphoma Patients Using a Machine Learning Model
Source: Vet Sci. 2021 Dec 2;8(12):301. doi: 10.3390/vetsci8120301 (PMC8704313; doi:10.3390/vetsci8120301)
Supplement: Supplementary file 1 [file vetsci-08-00301-s001.zip › vetsci-1485580-supplementary.pdf]

Supporting Information

Predicting dynamic clinical outcomes of the chemotherapy for canine lymphoma patients using a machine learning model

Jamin Koo[1,2,3], Kyucheol Choi[2], Peter Lee[1], Amanda Polley[1], Raghavendra Sumanth Pudupakam[1], Josephine Tsang[1], Elmer Fernandez[1], Enyang James Han[1], Stanley Park[1], Deanna Swartzfager[1], Nicholas Seah Xi Qi[1], Melody Jung[1], Mary Ocnean[1], Hyun Uk Kim[4], Sungwon Lim[1,2,*]

[1]ImpriMed Inc., 4030 Fabian Way, Palo Alto, CA 94303, USA
[2]ImpriMedKorea Inc., Seoul Startup Hub, Seoul 04147, Korea
[3]Department of Chemical Engineering, Hongik University, Seoul 04066, Korea
[4]Department of Chemical and Biomolecular Engineering, Korea Advanced Institute of Science and Technology (KAIST), Daejeon 34141, Korea

*To whom correspondence should be addressed: Sungwon Lim
Email: sungwon@imprimedicine.com

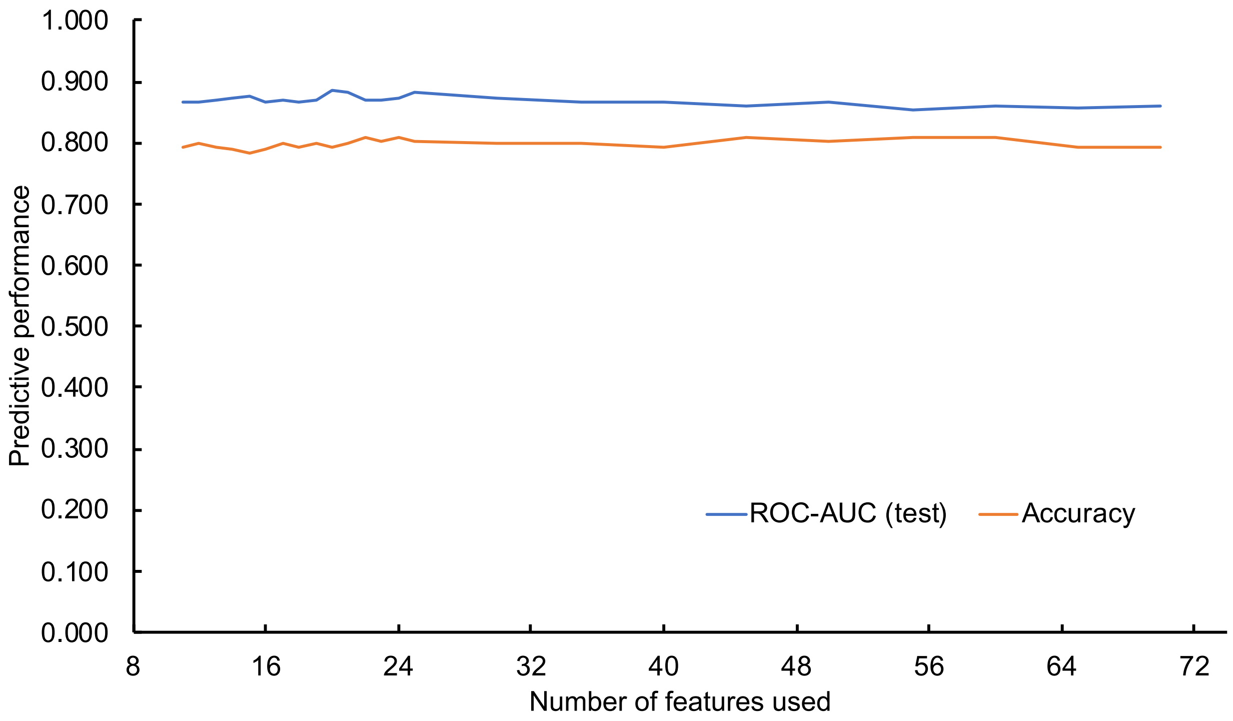

Fig. S1. Changes in the predictive performance of the RF model when the number of selected features *k* is increased from 7 to all. The top *k* features were chosen by the mutual information classification algorithm using the nearest neighbors method.

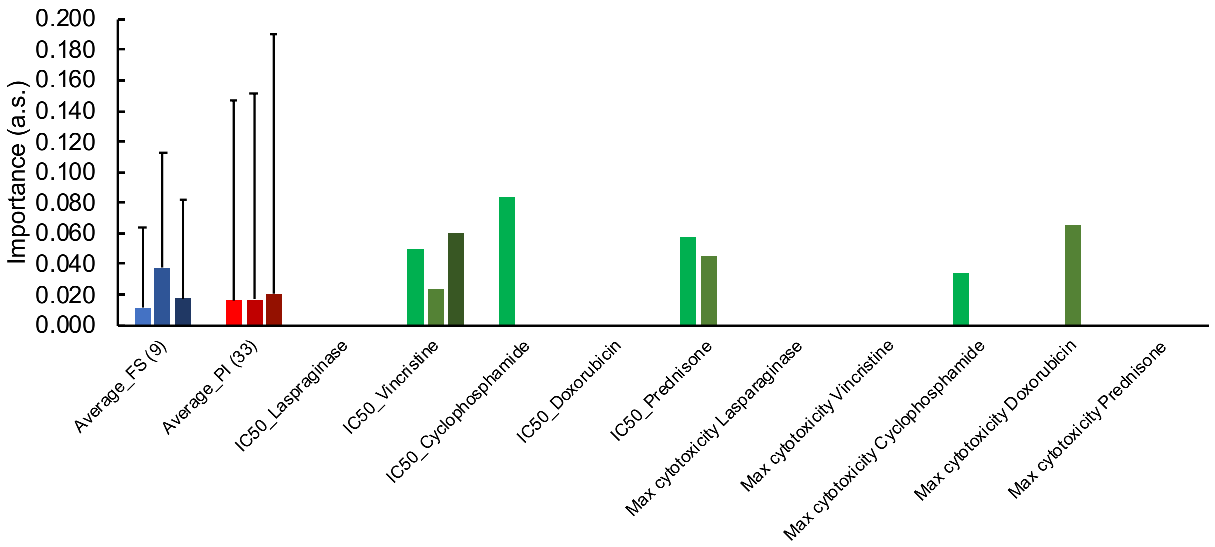

Fig. S2. Importance of the features to the ML models predicting clinical outcome by the 4th, 8th, and 12th week since the initiation of the chemotherapy. The bars represent the importance computed for the models exhibiting the highest predictive performance. For each feature, three bars are drawn that represent the importance for the models predicting clinical outcome by the 4th, 8th, and 12th week (left to right). Instead of showing the importance of every single feature, the average and maximum (represented by the error bar) of all FC and PI data are shown (blue and red colored bars on the left side). In contrast, the importance of each feature (green colored bars) is shown individually for the DS data. As one can see, the number of features selected decreased from 4 to 1 for the DS data when predicting clinical outcome for the 4th vs. 12th week. This is the opposite of the trend observed for the PI data, for which the average and maximum importance increased for the later time points.

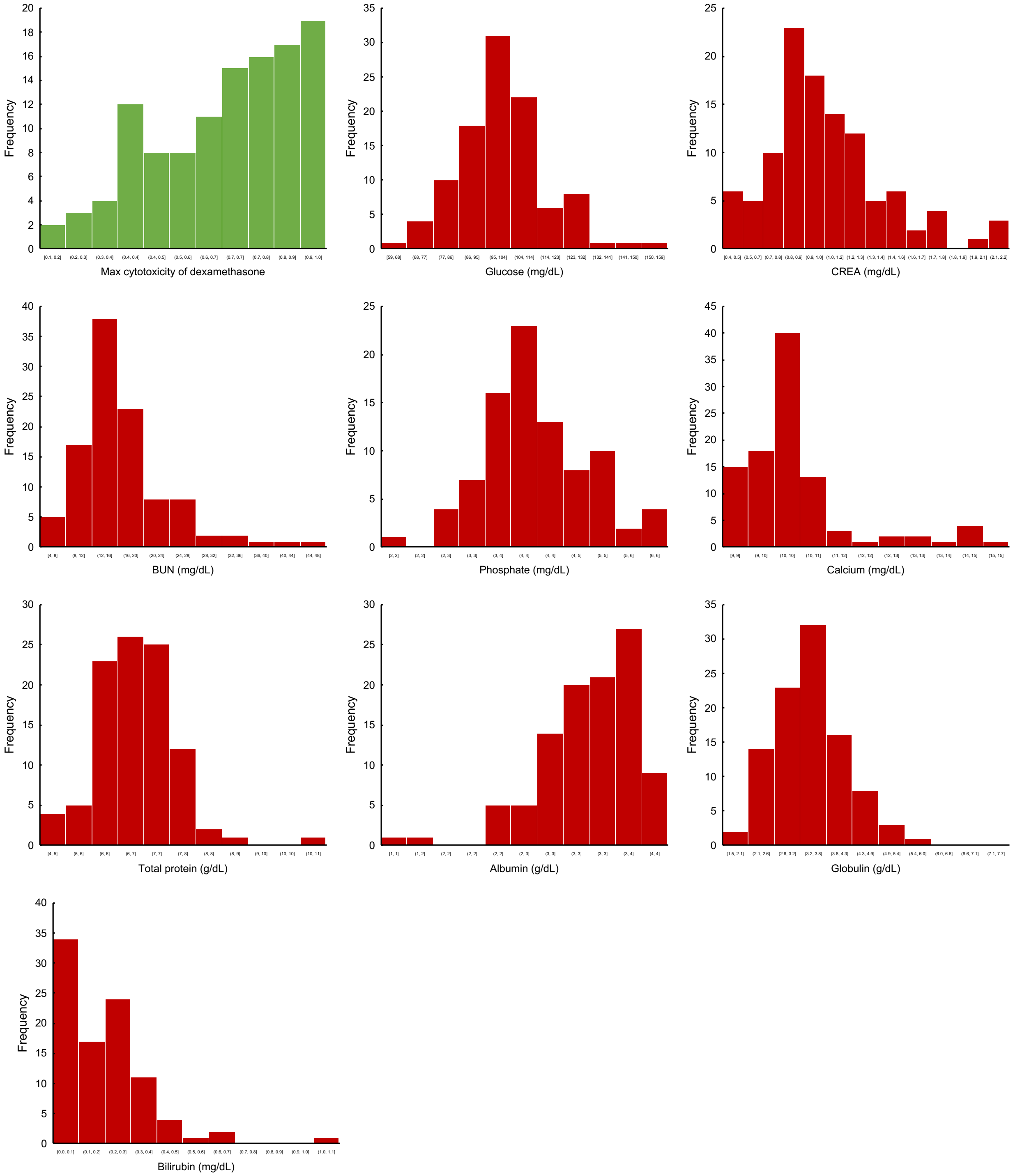

Fig. S3. Distribution of the raw data for the features having high (> 50%) percentages of missing values. The green and red colors of bars indicate that these features are part of the DS or PI data, respectively.

Table S1. List of the 8 additional chemotherapeutic drugs for canine lymphoma considered in this study.

Drug	Type
Mitoxantrone	Anthracenedione antineoplastic agent
Vinblastine	Vinca alkaloid that inhibits the assembly of microtubules
Actinomycin	Transcription inhibitor
Rabacfosadine	Nucleotide analog
Chlorambucil	Alkylating agent blocking the formation of DNA and RNA
Mechlorethamine	Alkylating agent disrupting DNA replication
Lomustine	Alkylating agent disrupting DNA replication
Melphalan	Alkylating agent blocking the formation of DNA and RNA

Table S2. List of the 33 features comprising the PI data.

Feature	Description	Feature	Description
Age	age at the time of diagnosis	Lymp	lymphocyte count (K/μL)
Sex	male or female	Mono	monocytes count (K/μL)
Breed	retriever, collie, poodle, mix, etc.	EOS	eosinophil count (K/μL)
IsRelapse	relapse or naïve	Baso	basophil count (K/μL)
Stage	extent of spread of tumor (1-5)	Glu	blood glucose level (mg/dL)
Substage	how patients feel (a or b)	CREA	blood creatine level (mg/dL)
Type	B, T, or other	BUN	blood urea nitrogen level (mg/dL)
Grade	growth rate of tumor	Phos	blood phosphorus level (mg/dL)
WBC	white blood cell count (K/μL)	Ca	blood calcium level (mg/dL)
RBC	red blood cell count (M/μL)	TP	blood total protein level (g/dL)
HGB	hemoglobin content (g/dL)	Alb	blood albumin level (g/dL)
HCT	proportion of RBC (%)	Glob	blood globulin level (g/dL)
MCV	mean corpuscular volume (fL)	Alt	alanine aminotransferase level (U/L)
MCH	mean corpuscular hemoglobin (pg)	Alkp	alkaline phosphatase level (U/L)
MCHC	mean corpuscular hemoglobin concentration (%)	GGT	gamma-glutamyl transpeptidase level (U/L)
PLT	average number of platelets (K/μL)	Tbil	total bilirubin level (mg/dL)
Neu	neutrophil count (K/μL)		

Table S3. Performance of the other ML models when predicting clinical outcome across the time points.

ML method	ROC-AUC (cross-validation)		
	4th week	8th week	12th week
Random Forest	0.841	0.893	0.823
Support Vector Machine	0.830	0.739	0.659
Linear Regression	0.831	0.782	0.681

Table S4. Grid of hyperparameters used to optimize the performance of the RF models.

Hyperparameter	Grid
n_estimators	30, 50, 100
max_features	auto, sqrt, log2
max_depth	6, 7, 8, 9
criterion	gini, entropy
min_ samples_leaf	3, 4, 5
